# Neural Plasticity in the Brain during Neuropathic Pain

**DOI:** 10.3390/biomedicines9060624

**Published:** 2021-05-31

**Authors:** Myeong Seong Bak, Haney Park, Sun Kwang Kim

**Affiliations:** 1 Department of Science in Korean Medicine, Graduate School, Kyung Hee University, Seoul 02447, Korea; msbak@khu.ac.kr (M.S.B.); haney9@khu.ac.kr (H.P.); 2 Department of Physiology, College of Korean Medicine, Kyung Hee University, Seoul 02447, Korea

**Keywords:** neuropathic pain, neural plasticity, primary somatosensory cortex, anterior cingulate cortex, periaqueductal grey, basal ganglia

## Abstract

Neuropathic pain is an intractable chronic pain, caused by damage to the somatosensory nervous system. To date, treatment for neuropathic pain has limited effects. For the development of efficient therapeutic methods, it is essential to fully understand the pathological mechanisms of neuropathic pain. Besides abnormal sensitization in the periphery and spinal cord, accumulating evidence suggests that neural plasticity in the brain is also critical for the development and maintenance of this pain. Recent technological advances in the measurement and manipulation of neuronal activity allow us to understand maladaptive plastic changes in the brain during neuropathic pain more precisely and modulate brain activity to reverse pain states at the preclinical and clinical levels. In this review paper, we discuss the current understanding of pathological neural plasticity in the four pain-related brain areas: the primary somatosensory cortex, the anterior cingulate cortex, the periaqueductal gray, and the basal ganglia. We also discuss potential treatments for neuropathic pain based on the modulation of neural plasticity in these brain areas.

## 1. Introduction

Pain is an unpleasant sensory and emotional experience associated with, or resembling that associated with, actual or potential tissue damage [1]. Neuropathic pain, defined as pain caused by a lesion or disease of the somatosensory nervous system [2], can be induced by factors such as infection, nerve injury, chemotherapy, and diabetes [3,4,5]. Pain symptoms are characterized by spontaneous pain, mechanical or thermal allodynia, and hyperalgesia to stimuli [6]. Approximately 7–10% of the general population suffer from neuropathic pain [7].

Despite the development of numerous analgesics based on pain studies at the peripheral and spinal cord levels, they still have limited effects on neuropathic pain [8]. Analgesic drugs currently being applied in clinical practice for neuropathic pain have serious adverse effects, such as depression, memory loss, nystagmus, and addiction [9,10]. Their lack of effectiveness is probably due to an insufficient understanding of the mechanisms underlying neuropathic pain [11]. Neural plasticity in the brain has been reported to be involved in the development and maintenance of neuropathic pain [12]. Therefore, to fully understand the mechanism underlying neuropathic pain and to develop a satisfactory treatment strategy, it is important to elucidate neural plasticity in the brain during neuropathic pain.

Neuropathic pain is accompanied by systemic plastic changes in the brain. This neural plasticity ranges from functional to structural changes in neurons. Functional changes include alternations in the calcium activity of neurons, excitatory postsynaptic current (EPSC) frequency, EPSC amplitude, intrinsic excitability, synaptic strength, and brain oscillations [13,14,15,16]. Structural changes include increased turnover rate or density of dendritic spines in the S1 and ACC and gain of presynaptic axonal boutons in the S1 [7,16,17]. These changes can serve as biomarkers for neuropathic pain. Additionally, reversal of neural plasticity leads to analgesic effects, indicating that this modulation can be a potential therapeutic method for neuropathic pain.

Pathological neural plasticity during neuropathic pain has been observed in several brain areas. In particular, cortical areas such as the primary somatosensory cortex (S1) and the anterior cingulate cortex (ACC) have been frequently reported to be associated with neuropathic pain [14,17]. These regions are known to play essential roles in pain sensation and pain affect, respectively. The periaqueductal gray (PAG), a key area of descending pain control, has also been studied extensively [18]. Although the role of basal ganglia in pain mechanisms has not been a focus of research [19], recent studies suggest that the basal ganglia have a unique role in neuropathic pain. In this review paper, we discuss neural plasticity in the above four areas of the brain during neuropathic pain and potential treatments based on the modulation of neural plasticity in each brain area.

## 2. The Primary Somatosensory Cortex

Neurons in the S1 play a role in the signal processing of various types of mechanosensation with feature-dependent response patterns at the single-cell or population level [20]. In a broad range of species, including humans, an association between neural activity in the S1 and pain processing has been reported. In humans, functional brain imaging [21] and magnetoencephalography (MEG) studies [22] demonstrated that peripheral nociceptive stimuli activate S1 neurons. An imaging study focused on the intensity coding of nociceptive stimuli in humans and found a high correlation between the stimulus intensity and the S1 activity [23]. Monkey studies also found that information about the intensity of nociceptive stimuli is encoded in S1 neurons [24]. The mice who had lesions on the hindlimb S1 region showed loss of mechanical allodynia induced by hind paw inflammation, but this did not attenuate the inflammation-induced paw volume changes or pain affect as demonstrated by place avoidance test [25]. These results indicate that S1 plays a critical role in the processing of pain sensation. Interestingly, however, recent studies have shown that S1 functions are not restricted to pain sensation but are associated with other aspects of pain, such as pain affect and anxiety. S1 has a role in the comorbid anxiety symptoms of pain [26], and projecting neurons in the S1 into the ACC play an important role in affective pain signals [27].

Cortical areas, including S1, receive nociceptive signals through the lamina I spino-thalamo-cortical circuit. Pain signals originating from laminae I neurons in the spinal dorsal horn are delivered to the S1 via the contralateral thalamus [28,29,30]. Additionally, the spino-parabrachial-thalamic pathway has also been suggested for the ascending pain pathway [31]. A recent study showed direct evidence for the spino–parabrachial–thalamic pathway in nociception [32]. However, the association between this pathway and cortical areas requires further verification.

### 2.1. Neural Plasticity in the S1 during Neuropathic Pain

Human studies have demonstrated that the activity of S1 is closely associated with the development and maintenance of chronic pain. Functional magnetic resonance imaging (fMRI) studies have revealed that neuropathic patients display increased S1 neural activity in response to painful [33] or allodynic stimuli [34].

In mice, nerve injury alters several protein activities, and it is eventually linked with neural plasticity in the S1. Upregulation of metabotropic glutamate receptor 5 (mGluR5) signaling in S1 astrocytes and synaptogenic thrombospondin 1 (TSP-1) release from astrocytes appeared after partial sciatic nerve ligation (PSL) injury. These changes in protein activity lead to the increase in dendritic spine turnover of the S1 pyramidal neurons. Notably, microinjection of TSP-1 into the S1 of control mice increased dendritic spine turnover rate and caused mechanical hypersensitivity that lasted for at least 1 month. This result indicates that pain could be induced by functional and structural changes in S1 without changes in the spinal cord or peripheral nerves, and that activation of S1 astrocytes plays a role in neuropathic pain [35].

Another study also reported that the activation of astrocytes in S1 drives abnormal pain. Ishikawa et al. found that disinhibition of the ipsilateral side of S1 by microinjection of SR95531, a gamma-aminobutyric acid (GABA)-A receptor antagonist, in neuropathic pain mice undergoing PSL induced mirror image pain that does not appear in this neuropathic pain model without disinhibition. This mirror-image pain was blocked by the administration of fluoroacetate, an astrocytic Krebs cycle inhibitor, or by knockout of *IP_3_R2* genes [36].

With structural changes in postsynaptic dendritic spines during neuropathic pain, the presynaptic components of neurons are also altered during neuropathic pain. Nerve injury leads to an increase in gain rate of axonal boutons. Notably, bouton turnover in S1 appeared only in the development phase of neuropathic pain, and it is correlated with changes in spine morphology. Thus, it has been suggested that plasticity of presynaptic and postsynaptic structures plays a role in the development of neuropathic pain [37].

Intrinsic plasticity, a type of neural plasticity, has also been reported in neuropathic pain. The transient spinal cord ischemia (tSCI) model mice showed bilateral mechanical allodynia and increased spontaneous calcium activity of pyramidal neurons in the S1. Electrophysiological study also showed an increased number of action potentials in response to current injections, increased spontaneous action potential firing rate, and increased EPSP frequency [38].

With the plasticity of excitatory neurons during neuropathic pain, inhibitory interneurons in the S1 showed neural plasticity during neuropathic pain, but the direction of the modulation was heterogeneous depending on the subtype. Somatostatin (SOM)-expressing inhibitory neurons and parvalbumin (PV)-expressing inhibitory neurons showed decreased calcium activity after spared nerve injury (SNI), whereas vasoactive intestinal polypeptide (VPN)-expressing interneurons increased their activity. These changes synergistically move pyramidal neurons into hyperactivity [39]. Wei et al. consistently reported that chronic constrictive injury (CCI) leads to the same directional regulation of neural activity [40].

The plasticity of interneurons not only contributes to the hyperactivity of pyramidal neurons but also plays a key role in the regulation of neural oscillations. In electroencephalography (EEG) studies, gamma-band oscillations in the brain are correlated with pain responses [41,42]. Several studies have reported that mice undergoing capsaicin-induced pain or neuropathic pain show elevated gamma and alpha power in the S1 during the resting state, as well as increased gamma power in response to noxious stimuli [43,44]. Artificially inducing gamma oscillations via optogenetic activation of PV^+^ interneurons in S1 enhances nociceptive sensitivity and induces aversive avoidance behavior in naïve mice [45]. These results indicate that gamma oscillations in S1 are highly correlated with neuropathic pain accompanied by upregulation of gamma power in S1.

### 2.2. Experimental Manipulations to Reverse the Neural Plasticity

Manipulations of brain activity to reduce pain have been proposed based on neural plasticity of S1 during neuropathic pain. Reversal of protein activity after nerve injury ameliorates pain phenotypes in mice. Microinjection of 2-methyl-6-(phenylethynyl)-pyridine (MPEP), a mGluR5 antagonist, into the S1 reversed the overexpression of TSP-1 and alleviated mechanical allodynia. Knockdown of TSP-1 expression in S1 using siRNA also significantly reduced spine turnover rate of S1 neurons and alleviated mechanical allodynia [35]. Additionally, immediate peripheral nerve blockade by tetrodotoxin (TTX) after PSL injury also prevented structural synaptic plasticity in S1 and mechanical allodynia following nerve injury [46].

In contrast to the above results, Xiong et al. reported that the upregulation of S1 activity, but not inhibition, could lead to analgesic effects in neuropathic pain. They found that tSCI leads to transient downregulation of neural activity of the S1 six hours after surgery. Based on this observation, they applied optogenetic stimulation to unilateral S1, resulting in a significant decrease in S1 hyperexcitation and mechanical hypersensitivity [38].

Neuronal subtype-specific manipulation provides insights into neuropathic pain. Chemogenetic activation of SOM^+^ neurons in the early phase of neuropathic pain significantly reduced pyramidal neuron hyperactivity and mechanical hypersensitivity in SNI mice. This analgesic effect lasted for a month [39]. Additionally, electroacupuncture (EA) reversed the altered activity of pyramidal, SOM^+^, and VIP^+^ neurons after nerve injury and alleviated mechanical and thermal hypersensitivity. Activation of cannabinoid 1 (CB1) receptors is essential for this EA-induced analgesic effect, as demonstrated by co-administration of EA and AM251, a selective CB1 receptor antagonist [40].

Reducing theta oscillations in S1 could be suggested for the treatment of pain. The gamma oscillations of S1 are affected by thalamic input [47]. GABA-mediated tonic activation of inhibitory neurons in the thalamus is suppressed during chronic inflammatory pain [48]. Based on these reports, Lablanc et al. induced burst firing in the thalamus by activation of GABAergic neurons using optogenetic manipulation to silence theta oscillations in S1. Activation of GABAergic neurons in the thalamus alleviated the increased theta oscillations in the S1 and reduced mechanical allodynia induced by capsaicin [49].

Complete Freund’s adjuvant (CFA) injection into the hind paw, which induces sub-chronic inflammatory pain in mice, leads to neural plasticity in the brain. Inhibition of the S1 activity results in analgesic effects in CFA pain models. Eto et al. showed that microinjection of CNQX, a competitive α-amino-3-hydroxy-5-methyl-4-isoxazolepropionic acid (AMPA) receptor antagonist, into S1 increased the paw withdrawal threshold and decreased the slope of the field excitatory postsynaptic potential (fEPSP) of the ACC in response to electrical stimulation of the mouse hind paw [50]. Notably, modulation was performed in the S1 area, but the ACC was affected. This result indicates that the ACC can be regulated by S1 neurons.

## 3. The Anterior Cingulate Cortex

The ACC plays a role in high levels of cognitive functions such as error detection [51], decision-making [52], emotion [53], and pain processing [54]. Early studies on pain found that the ACC is associated with pain affect [55,56,57] rather than pain sensation [58]. ACC lesions in rats who underwent nerve ligation did not change mechanical hypersensitivity but reduced escape/avoidance behavior, which reflects damage to the affective component of pain [59]. Recent studies have extended the role of the ACC in pain processing. They reported that ACC has roles in several dimensions of pain, such as pain sensation [27,60], anticipation [61], and social transfer of pain [62].

ACC pyramidal neurons receive pain inputs from the thalamus and the S1. The output projections of the ACC are connected to the PAG, which plays an important role in pain modulation at the spinal cord level via the descending inhibitory circuits [63]. Functional connectivity analysis showed a significant correlation between ACC and PAG in pain states [64]. Additionally, neurons in the ACC project to the insular cortex [65] or dorsal horn of the spinal cord, and these projections play a role in the top–down system for rapid modulation of spinal sensory transmission [66].

### 3.1. Neural Plasticity in the ACC during Neuropathic Pain

Neuropathic pain is accompanied by changes in the level of several proteins related to synaptic plasticity in the ACC. Neural cell adhesion molecule 1 (NCAM1), which plays a role in neurite outgrowth and spine formation, showed an increased turnover rate after nerve injury in mice. This change in the expression level of NCAM1 caused changes in spine morphology in the ACC, which was demonstrated by NCAM1 knockdown with adeno-associated virus (AAV) that expresses shNCAM1. Morphological changes are also reflected in the increased miniature excitatory postsynaptic current (mEPSC) amplitudes of pyramidal neurons in the ACC [67].

PKMζ, a key molecule for maintaining long-term potentiation (LTP), plays a role in neuropathic pain-associated neural plasticity in the ACC. The expression levels of PKMζ and p-PKMζ in the ACC increased significantly after common peroneal nerve (CPN) ligation in mice. PKMζ maintains pain-induced LTP and increases the amplitudes of evoked EPSCs in neuropathic pain. Knock-out of calmodulin-stimulated adenylyl cyclase-1 (AC1), which acts upstream of PKMζ, reversed the upregulation of PKMζ and p-PKMζ and suppressed mechanical allodynia after nerve injury [68].

Using multiple whole-cell recordings in layer 5 neurons of the ACC in mice undergoing CCI, Blom et al. investigated the plasticity mechanisms that occur in neural circuits in the ACC during neuropathic pain. They found a loss of functional connections between excitatory and inhibitory neurons as well as a decrease in mEPSC and miniature inhibitory postsynaptic current (mIPSC) frequencies. There were no significant changes in synaptic properties of the remaining connected pairs. In addition, they found that CCI potentiates the intrinsic excitability of pyramidal neurons, but not that of inhibitory neurons [69].

Using in vivo two-photon calcium imaging, Zhao et al. showed that layer 5 pyramidal neurons in mouse ACC showed enhanced calcium activity in the absence or presence of pain stimuli in SNI-induced neuropathic pain [70]. In addition, an electrophysiological study in mouse brain slices also reported the increased spontaneous EPSC frequency and the elevated intrinsic excitability of layer 2/3 pyramidal neurons in the ACC after SNI [71].

### 3.2. Modulation of Neural Plasticity in the ACC

Accumulating evidence indicates that the ACC exhibits hyperactivity in neuropathic pain, which is closely associated with LTP [72]. Therefore, it was hypothesized that LTP in the ACC is essential for the maintenance of neuropathic pain, and by reversing LTP to the baseline, analgesic effects may be observed. Li et al. demonstrated this hypothesis using z-pseudosubstrate inhibitory peptide (ZIP), which blocks the maintenance of LTP.

Microinjection of ZIP into the ACC leads to a significant analgesic effect demonstrated by the mechanical allodynia test and the place preference test [68] in mice with CPN ligation. The increase in the EPSC amplitudes of pyramidal neurons in the ACC in these neuropathic pain mice was also reversed by ZIP. Although there are promising results that ZIP is not an inhibitor for PKMζ [73,74], ZIP still reverses LTP. As another approach, Ko et al. induced analgesic effects by injecting anisomycin into the ACC of neuropathic pain model mice. Anisomycin, which is a protein synthesis inhibitor, blocks LTP formation by inhibiting the synthesis of plasticity-related proteins. Infusion of anisomycin into the ACC reversed the increased spine turnover rate, mechanical allodynia, and thermal allodynia [67].

mTOR is a serine-threonine protein kinase that regulates synaptic plasticity by activating synaptic protein synthesis [75]. Thus, the inactivation of mTOR activity may reverse LTP in neuropathic pain. Um et al. demonstrated this hypothesis by microinjection of rapamycin into the ACC. They found that the injection of rapamycin led to the reduction in mechanical allodynia and in co-localization of PSD-95 and GluA1 in the ACC of neuropathic pain rats. Additionally, the neural activity in the ACC in response to peripheral electrical stimulation was enhanced in neuropathic pain, and this ACC activation was reduced by rapamycin [76]. Understanding neuropathic pain as a concept of memory is intriguing. However, the trials described above have been limited to short-term analgesic effects. Since electrophysiology experiments on brain slices from the ACC of neuropathic pain mice consistently report an increase in intrinsic excitability, a method involving modulation of both synaptic plasticity and intrinsic excitability can be suggested for further study.

Indoleamine 2,3-dioxygenase 1 (IDO1) is a rate-limiting enzyme in tryptophan metabolism, and it has been linked to neuropathic pain [77]. The expression level of IDO1 increased in the ACC of rats receiving spinal nerve ligation (SNL). Oral administration of PCC0208009, an IDO1 inhibitor, attenuated mechanical allodynia and thermal hyperalgesia and induced conditioned place preference (CPP) in SNL animals. PCC0208009 also reversed the increase in dendritic spine density and suppressed the upregulation of phosphorylation level of N-methyl-D-aspartate receptor (NMDA)-2B receptors in the ACC after nerve injury. These results indicate that PCC0208009 can alleviate neuropathic pain by regulating synaptic plasticity in the ACC [78].

It has been reported that direct manipulation of neural activity in the mouse ACC can induce analgesic effects in CFA-induced inflammatory pain. Optogenetic inhibition of excitatory pyramidal neurons in the ACC induced CPP. Moreover, chemogenetic suppression of excitatory pyramidal neurons in the ACC alleviated mechanical hyperalgesia [79].

## 4. The Periaqueductal Gray

The PAG is the core region for endogenous descending pain modulation [80]. The descending pathway originates within the PAG and projects to the spinal dorsal horn through the rostral ventromedial medulla (RVM) and the locus coeruleus (LC). Activation of this descending system elicits analgesic effects by inhibiting ascending nociceptive signal transmission at the spinal cord [81]. Serotonin from the RVM and noradrenalin from the LC to the spinal dorsal horn are major outputs for this pain modulation system [82].

Recent studies have reported that connections of the PAG to other brain regions undergo plastic changes after nerve injury, and these changes are strongly associated with the development of neuropathic pain. Projecting neurons in the central medial (CeM) amygdala to the PAG undergo plastic changes due to inflammatory pain [83]. Connections between the PAG and the mPFC also showed changes in functional connectivity in neuropathic pain. Cheriyan et al. reported that cortico-PAG neurons showed a significant reduction in intrinsic excitability in brain slices of mice with neuropathic pain induced by CCI [84]. Huang et al. identified a long-range brain circuit that is crucial for pain processing. Using optogenetic approaches combined with pharmacology, they revealed that the basolateral amygdala (BLA)–PFC–PAG circuits regulate neuropathic pain behaviors, such as mechanical and thermal hypersensitivity. SNI enhances synaptic inputs from the BLA to inhibitory neurons in the mPFC, resulting in decreased analgesic modulation by inhibiting projections from the mPFC to the PAG [8].

### 4.1. Changes in Opioid Sensitivity in the PAG during Neuropathic Pain

The μ-opioid receptor (MOR) in the PAG contributes to the analgesic effects mediated by opioids [82,85], supported by the fact that the direct infusion of opioids to the PAG sufficiently induced analgesic effects [86]. The analgesic efficacy of opioids is reduced in neuropathic pain, but the underlying mechanism is still elusive [11]. Neural plasticity of the PAG after nerve injury may be related to these reduced analgesic effects.

Hoot et al. reported that CCI in mice reduced in [D-Ala^2^, N-MePhe^4^, Gly-ol]-enkephalin (DAMGO)-stimulated GTPγS binding in the PAG, indicating that the adaptation of MOR-mediated G-protein activity and desensitization of the MOR [87]. Consistent with this report, Maarrawi et al. showed that human patients suffering from peripheral neuropathic pain have decreased opioid receptor binding activity, as demonstrated by PET imaging [88].

Activation of MOR decreases presynaptic GABA release in PAG neurons. Hahm et al. found that GABA release was upregulated after sacral nerve transection in rats, but the level of inhibitory effects on presynaptic GABA release by activation of MOR was intact in neuropathic pain rats [89]. Therefore, the actions of opioids and nerve injury on presynaptic GABA release could be considered mutually exclusive. However, the effect of opioids is sufficiently masked in neuropathic pain, resulting in a reduction in the analgesic effects of opioids.

Paradoxically, opioids can induce hyperalgesia. It is reported that activation of calmodulin-dependent kinase type II (CaMKIIα) mediates opioid-induced hyperalgesia (OIH). Inhibition of CaMKIIα activity in the PAG, CeLC, or the spinal cord by KN93, an inhibitor of CaMKIIα, reversed both mechanical and thermal allodynia in mice with OIH. The frequency and amplitude of mEPSCs in PAG cells were decreased by KN93 addition to PAG slices. These results suggest that CaMKIIα may modulate OIH via the CeLC-PAG-RVM-spinal-cord-descending facilitatory pain pathway [90].

### 4.2. Plasticity in Glutamatergic Pathway in the PAG during Neuropathic Pain

Nerve injury changes the properties of glutamate receptors in PAG neurons, resulting in reduced activity and malfunction of descending pain inhibition. The PAG showed consistent mGluR1/5 and calcium activity in the normal state to regulate the descending pain modulatory signal. The SNL in rats induced mechanical allodynia and decreased mGluR1/5 activity of PAG neurons. PAG-RVM-projecting neurons showed a reduction in excitatory input, as demonstrated by electrophysiological recordings of retrogradely labeled neurons. Interestingly, a single injection of mGluR5 inverse agonist 2-methyl-6-(phenylethynyl) pyridine (MPEP) or mGluR1 inverse agonist BAY 36-7620 in naïve animals resulted in mechanical allodynia and decreased the intrinsic excitability of ventrolateral PAG (vlPAG) neurons [91].

With metabotropic glutamate receptors, the properties of ionotropic glutamate receptors are also altered in neuropathic pain. Hu et al. investigated the role of NMDA receptor subunits in the PAG during persistent inflammatory pain. They found that injection of CFA into mouse hind paw caused upregulation of NR2B-containing NMDA receptors in the PAG, while NR2A-containing NMDA receptors were not altered. Whole-cell patch-clamp recordings revealed that NMDA receptor-mediated mEPSC amplitudes increased in the PAG [92].

Consistent with this report, Ho et al. showed that SNL resulted in upregulation of the NR1 and NR2B subunits in the rat vlPAG. Upregulation of NMDARs after nerve injury might lead to the hypofunction of AMPARs through subcellular redistribution. Neurons in brain slices from neuropathic rats have decreased EPSC frequency and amplitude. [93]. AC-cAMP-PKA signaling in the vlPAG may be associated with these changes in the functions of glutamate receptors. An AC activator forskolin-induced EPSC potentiation was impaired in the vlPAG of SNL-induced neuropathic pain rats. This impairment may contribute to the hypofunction of glutamatergic pathway in the PAG during neuropathic pain [94].

Prevention or reversal of neural plasticity in the PAG could induce analgesic effects in chronic pain conditions. Chung et al. prevented the chronification of neuropathic pain by inhibiting Homer1a, an intracellular interacting molecule that is expressed activity-dependently. Homer1a is known to regulate mGluR5 activity [95], and its transcriptional level increased in the PAG after SNL. Inhibition of Homer1a translation by shHomer1a decreased mechanical allodynia in rats. Notably, this modulation of Homer1a specifically affects the late phase of neuropathic pain, and this analgesic effect lasts for a long time. They also showed that administration of 3,5-dihydroxyphenylglycine (DHPG), the mGluR1/5 agonist, into the PAG leads to an analgesic effect on SNL-induced mechanical allodynia [91].

Hu et al. showed that microinjection of Ro 25-6981, a NR2B antagonist, into the PAG inhibited thermal hyperalgesia bilaterally in rats with CFA-induced inflammatory pain. Hyperoside, a flavonoid compound isolated from Rhododendron ponticum L., suppressed the upregulation of NR2B-containing NMDA receptors in the PAG after CFA and led to an analgesic effect in CFA-injected mice [92]. Ho et al. reported that microinjection of forskolin into the vlPAG alleviates SNL-induced mechanical allodynia in rats, suggesting that activation of AC-cAMP-PKA signaling in the PAG can lead to an analgesic effect via glutamatergic synaptic plasticity [94].

## 5. The Basal Ganglia

The basal ganglia consist of several nuclei: the striatum (the caudate nucleus, the putamen, and the nucleus accumbens core (NAc)), the external globus pallidus (GPe), the internal globus pallidus (GPi), the subthalamic nucleus (STN), and the substantia nigra (SN) [96]. The midbrain dopaminergic (DA) neurons are associated with reward, cognition, and motor control, which are mediated by the mesolimbic (ventral tegmental area, VTA), mesocortical (VTA-retrorubral), and nigrostriatal (SN pars compacta, SNc) pathways, respectively. Therefore, DA cells are an integral part of the basal ganglia [97].

Although best known for their role in motor systems, the basal ganglia are a major site for adaptive plasticity in the brain, affecting a broad range of normal behaviors and neurological and psychiatric conditions. The basal ganglia integrate incoming nociceptive information to contribute to coordinated, graded motor responses in complex and spatially guided pain avoidance/nocifensive behaviors [98].

Previous studies have suggested not only the role of the basal ganglia in nociceptive sensorimotor integration, but also the potential nociceptive pathways into and out of the basal ganglia [99]. The basal ganglia receive direct and indirect nociceptive inputs from the spinal cord and the thalamus, respectively. Cortical regions, such as ACC and S1, also send pain-associated signals to the basal ganglia, which contributes to the cortico-basal ganglia-thalamic loop that integrates multiple information of pain, including sensory, motor, emotional, cognitive, and autonomic components [96].

### Neural Plasticity in the Basal Ganglia during Neuropathic Pain and Its Modulation

Substance P (SP) is a well-known neuropeptide involved in the transmission of nociceptive information in the spinal cord [100], but, interestingly, SP showed the opposite effect in the striatum. Nakamura et al. found that the infusion of SP into the striatum inhibited PSL-evoked mechanical hypersensitivity, which was blocked by the co-infusion of NK1 receptor antagonist CP96345. Co-infusion of atropine, but not mecamylamine, also blocked the analgesic effect of SP infusion into the striatum, suggesting that activation of striatal muscarinic acetylcholine receptors through NK1 receptors could be a potential therapeutic method for neuropathic pain [101].

In addition to the DA inputs, the striatum also highly expresses the endogenous opioid molecules and receptors. In the NAc of CCI mice, the mRNA levels of κ and δ opioid receptors as well as prodynorphin and proenkephalin were significantly upregulated. These observations suggest that endogenous opioid signaling within the NAc may be strengthened in neuropathic pain conditions [102].

Galanin plays an important role in the regulation of nociceptive information at the periphery and spinal cord levels [103]. Interestingly, galanin in the NAc also has an analgesic effect. In the NAc of PSL rats, the expression of galanin receptor (GalR) 1 was upregulated. Local injection of GalR1 agonist M617 or galanin into the NAc attenuated mechanical and thermal hypersensitivity, which was blocked by GalR1/2 antagonist M35 [104].

The VTA may regulate pain-associated behaviors via a DA enhancement of mPFC output. Huang et al. showed that optogenetic stimulation of VTA DA terminals in the mPFC induces CPP as well as decreases mechanical hypersensitivity in SNI mice. The DA inputs increased the activity of mPFC neurons projecting to the vlPAG [105].

The VTA-NAc reward pathway plays a key role in modulating nociceptive information. Zhang et al. found that CCI leads to hyperactivation of contralateral VTA-NAc DA neurons and increases BDNF expression in the contralateral NAc. Optogenetic or pharmacological inhibition of these DA neurons reversed the overexpression of BDNF and thermal hyperalgesia after nerve injury. Conditional knockdown of BDNF in the VTA-NAc pathway or microinjection of anisomycin into the VTA to inhibit BDNF synthesis ameliorated thermal hyperalgesia in CCI mice [106].

Sirtuin 1 (SIRT1), which is a class III histone deacetylase, has been reported to alleviate neuropathic pain in the dorsal root ganglia and spinal cord. Li et al. found that SIRT1 was downregulated in the contralateral VTA of CCI mice. Thermal hyperalgesia and the elevation of Fos expression in both the VTA and NAc following nerve injury was inhibited by microinjection of SRT1720, an activator of SIRT1, into the VTA [107].

Plastic changes in the DA pathway of the basal ganglia during neuropathic pain are involved in the comorbidities of neuropathic pain. Two weeks following SNI in rats, the burst firing of VTA DA neurons was enhanced. In addition, extracellular dopamine levels increased, whereas the expression level of D2, but not D1, receptors decreased in the NAc of SNI animals. Another study also reported that the level of dopamine release within NAc in response to pain relief by pregabalin or a reward by sucrose intake increased during the early, but not late, phase of neuropathic pain in SNL rats. These alterations and dysfunction of the basal ganglia DA circuits may lead to comorbidities of neuropathic pain, such as depression and anxiety [108,109].

Pain is a prevalent non-motor symptom in Parkinson’s disease (PD), and STN is known to be closely associated with this pain. In PD model rats induced by injection of 6-hydroxydopamine (6-OHDA) into the SNc, STN cells exhibited longer responses with greater amplitude in response to painful foot shock stimuli, indicating that abnormal neural plasticity in the STN could mediate the pain symptoms in PD [110]. Another study also reported the hyperactivity of STN neurons and pain hypersensitivity in 6-OHDA-induced PD model mice, both of which were blocked by optogenetic inhibition of STN neurons. Interestingly, optogenetic inhibition of STN-SNr projections attenuated both mechanical and thermal hypersensitivity, while optogenetic inhibition of STN-GPi or STN-ventral pallidum (VP) projections attenuated only mechanical hypersensitivity in PD mice. These findings suggest that direct inhibition of STN neurons may be a potential therapeutic method to alleviate diverse pain symptoms in PD [111].

## 6. Conclusions and Perspectives

Both peripheral and central components of the pain transmission pathway showed tremendous plasticity, enhancing or reducing pain signals. When the plasticity facilitates protective reflexes, it can be beneficial, but when the changes persist, it may induce chronic pain conditions [112]. Recent development of technological tools, such as optogenetics, chemogenetics, and brain imaging, in rodent models allows us to understand the relationship between the regulation of neurotransmitters in the brain and chronic pain conditions [113].

**Figure 1:**
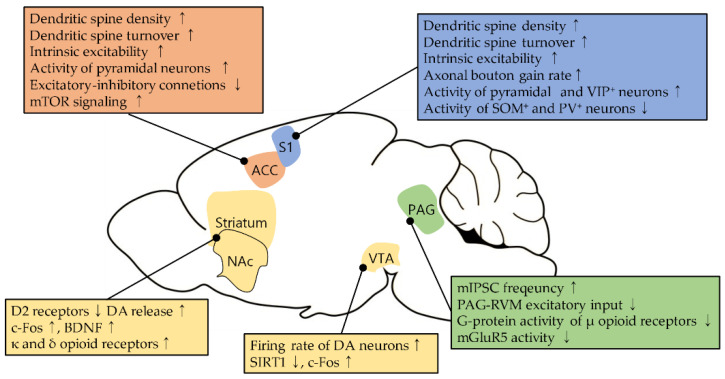
Neural plasticity in four brain areas during neuropathic pain. Arrows up (↑) and down (↓) indicate ‘increase’ and ‘decrease’, respectively.

Here, we summarized neural plasticity in four brain areas during neuropathic pain and modulations to reverse pathological states (Figure 1, Table 1). The most prominent manifestations of structural plasticity, such as dendritic spine turnover and changes in spine morphology, were observed in the S1 and the ACC. Functional plasticity in these two regions varies in excitatory and inhibitory neurons for each subtype, and it collectively shifts pyramidal neurons to hyperactivity. In contrast, the PAG showed decreased neural activity after nerve injury. The plastic changes in glutamatergic pathway, including the dysfunction of mGluR5 signaling, are involved in this hypoactivity. In addition, opioid signaling is altered in the PAG during neuropathic pain. These alterations may be associated with a reduction in the analgesic effects of opioids on neuropathic pain. The basal ganglia have been less focused in pain research. However, growing evidence suggests that they are also involved in the development and maintenance of neuropathic pain as well as comorbidities of neuropathic pain.

Pain is a multi-dimensional experience [114]. The S1 and ACC are critical regions for processing the sensory and emotional aspects of pain, respectively. The cognitive aspect is also an essential part of the pain experience. The mPFC plays a major role in the cognition of pain [115], and several studies have reported that the mPFC shows neural plasticity during neuropathic pain [116,117]. These changes in neural properties have complexities that appear in opposite effects depending on the sub-regions or layers of the mPFC [84]. This complexity makes it difficult to understand the role of mPFC in neuropathic pain. Nevertheless, connections with major pain processing areas [118] indicate the importance of the mPFC in understanding the mechanisms of neuropathic pain.

The PAG sends pain modulation signals to the rostroventromedial medulla (RVM) via its reciprocal connections [119]. The RVM also receives signals from the parabrachial nucleus and thalamus, and is considered to be the final relay in the descending pain modulatory system [120]. Moreover, as the RVM receives inputs from higher cortical sites, this region also provides a homeostatic mechanism that tones down or augments nociceptive inputs [121]. The RVM bidirectionally modulates pain through “on cells” and “off cells” that project to the spinal dorsal horn as well as the trigeminal nucleus caudalis [122,123]. The connectivity with pain processing areas implicates the importance of further study on neural plasticity in the RVM during neuropathic pain.

The basal ganglia are closely associated with PD by modulation of dopamine release [124]. More than 50% of PD patients suffer from pain, but the cause of this pain is still elusive [125,126]. In this review paper, we summarized the relationship between neural plasticity in the basal ganglia and neuropathic pain, which may contribute to the understanding of pain in PD patients. Indeed, structural and functional neural changes in the basal ganglia are thought to be related to the pain symptoms in PD patients.

Other brain regions in the limbic system are also involved in neuropathic pain. The amygdala is critical for the emotional aspect of pain [127] and showed neural plasticity in neuropathic pain. Parabrachial nucleus-central amygdala synapse was potentiated after nerve injury, and this synaptic plasticity is independent of NMDA receptors [128]. Interestingly, a study reported that the generation of new neurons in the amygdala was promoted during neuropathic pain [129]. The hippocampus displayed changes in dendritic spine morphology and neurogenesis rates following nerve injury [130,131]. These alterations are related to several molecules, such as tumor necrosis factor-alpha [132] and glycogen synthase kinase-3 beta [133]. Neural plasticity with the disruption of these proteins in the hippocampus is also associated with comorbidity of neuropathic pain, including anxiety and depression [131,134,135].

In this review, we discussed the potential treatments based on the modulation of neural plasticity in the brain of animals. Recently, with the advances in the field of the brain–computer interface, new techniques for assessing and stimulating the brain have emerged [136,137]. The brain stimulation techniques indeed have potential in clinical chronic pain treatment: transcranial magnetic stimulation in the motor cortex [138] and deep brain stimulation in the ACC [139], the PAG [140], and the subthalamic nucleus [141]. This technological progress may provide opportunities for the clinical transfer of preclinical brain modulation for the treatment of chronic neuropathic pain.

## Figures and Tables

**Table 1:** Neural plasticity in the brain during neuropathic pain and its modulation.

Area	Pain Model	Pathological Neural Plasticity	Modulation (M) and Its Effect (E)	Reference
S1	PSL * in mice	Spine turnover rates ↑ Astrocytic calcium activity ↑ Extracellular glutamates ↑ TSP-1 ↑	**M**: Infusion of MPEP, BAPTA, or siTSP-1 *IP_3_R2* Knockout **E**: Spine turnover rate ↓; TSP-1 ↓ Mechanical allodynia ↓	[35]
		Evoked potential of the S1 ↑ Spine turnover rates ↑	**M**: Immediate local nerve blockade **E**: Spine turnover rate ↓ Mechanical allodynia ↓	[46]
		Mushroom spines ↓ Thin spines ↑ Gain rate of axonal boutons ↑		[37]
	Transient spinal cord ischemia in mice	Spontaneous AP firing ↑ Intrinsic excitability ↑ EPSP frequency ↑ Calcium activity of pyramidal neurons ↑	**M**: Optogenetic stimulation of pyramidal neurons **E**: Sensory-evoked potential ↓ Spontaneous firing of layer 5 pyramidal neurons ↓ Intrinsic excitability of layer 5 pyramidal neurons ↓ Bilateral mechanical allodynia ↓	[38]
	SNI * in mice	Calcium activity of pyramidal neurons ↑ Calcium activity of VIP^+^ interneurons ↑ Calcium activity of SOM^+^ interneurons ↓ Calcium activity of PV^+^ interneurons ↓	**M**: Chemogenetic activation of SOM^+^ interneurons **E**: Calcium activity of pyramidal neurons ↓ Mechanical allodynia ↓	[39]
	CCI * in mice	Calcium activity of pyramidal neurons ↑ Calcium activity of VIP^+^ interneurons ↑ Calcium activity of SOM^+^ interneurons ↓	**M**: EA* intervention to GB30* and GB34* E: Calcium activity of pyramidal neurons ↓ Calcium activity of SOM^+^ interneurons ↓ Calcium activity of VIP^+^ interneurons ↓ Mechanical hyperalgesia ↓, Thermal hyperalgesia ↓	[40]
ACC	CPN * ligation in mice	mEPSC amplitude of pyramidal neurons ↑ Spine turnover rate ↑ NCAM1 turnover rate ↑	**M**: Anisomycin into the ACC **E**: Spine turnover rate ↓ Mechanical allodynia ↓ Thermal allodynia ↓	[67]
	CPN * ligation in mice	Evoked EPSC amplitude ↑ PKMζ and p-PKMζ ↑	**M**: ZIP* into the ACC **E**: Evoked EPSC amplitude ↓ Mechanical allodynia ↓, CPP ↑	[68]
	CCI* in mice	Connections between excitatory and inhibitory neurons ↓ Intrinsic excitability of pyramidal neurons ↑ mEPSC and mIPSC frequency in layer 5↓		[69]
	SNI * in mice	Spontaneous and evoked calcium activity of pyramidal neurons in layer 5 ↑		[70]
	SNI * in mice	sEPCS frequency of pyramidal neurons in layer 2/3 ↑ Intrinsic excitability of pyramidal neurons ↑		[71]
	SNL* in rat	Dendritic spine density in the ACC ↑ IDO1 expression in the ACC ↑	**M**: Oral administration of PCC0208009* E: Mechanical allodynia ↓ Thermal hyperalgesia ↓ Conditioned place preference ↑	[78]
	SNI * in rat	mTOR signaling ↑	**M**: Rapamycin * into the ACC **E**: Mechanical allodynia ↓ PSD-95 ↓, Evoked activity of ACC ↓	[76]
PAG	CCI * in mice	G-protein activity of the μ-opioid receptor-protein in reponse to DAMGO ↓		[87]
	Sacral nerve transection in rat	Frequency of GABAergic mIPSCs ↑ Frequency of IPSC response to DAMGO is not altered		[89]
	SNL * in rat	mGluR5 activity ↓ Homer1a expression ↑ sEPSC frequency of PAG-RVM neurons↓ Intrinsic excitability of PAG neurons ↓	**M**: DHPG into the PAG or shHomer1a **E**: Mechanical allodynia ↓ mGluR5 activity ↑ intrinsic excitability of PAG neurons ↑	[91]
	SNL * in rat	EPSCs frequency and amplitude ↓ NR1 and NR2B subunits ↑		[93]
	SNL * in rat	Forskolin-induced EPSC potentiation ↓	**M**: Infusion of forskolin into the PAG **E**: Mechanical allodynia ↓	[94]
	CFA *	NR2B subunits ↑ mEPSCs amplitude ↑	**M**: Ro 25-6981 * into the PAG Oral administration of Hyperoside* E: Thermal allodynia ↓, NR2B subunits ↓	[92]
BG	PSL * in rats	Muscarinic cholinergic neurons through NK1 receptors ↑	**M**: Co-infusion of SP and CP96345* into the striatum Atropine and mecamylamine into the striatum **E**: Mechanical hyperalgesia ↓	[101]
	CCI * in mice	Prodynorphin and proenkephalin in the NAc↑ κ and δ opioid receptors in the NAc ↑		[102]
	PSL * in rat	GalR1 in the NAc ↑	**M**: M617* or galanin into the NAc **E**: Thermal and mechanical hyperalgesia ↓	[104]
	SNI * in mice	DA in the NAc ↑	**M**: Optogenetic stimulation of DA terminals **E**: Mechanical hyperalgesia ↓, CPP ↑ Calcium activity of mPFC ↑ c-Fos in layer 5 of mPFC ↑	[105]
	CCI * in mice	Firing rates of VTA DA neurons ↑ c-Fos in VTA-NAc DA neurons↑ BDNF in the NAc ↑	**M**: Conditional knockdown of BDNF in the VTA-NAc pathway, Microinfusion of anisomycin into the VTA **E**: Thermal hyperalgesia ↓	[106]
	CCI * in mice	SIRT1 in the VTA ↓ c-Fos in the VTA ↑	**M**: SRT1720* into the VTA **E**: Thermal hyperalgesia ↓	[107]
	SNI * in rat	Burst firing of VTA DA neurons ↑ D2 receptors in the NAc ↓ DA release in the NAc ↑		[108]
	SNL * in rat	DA release in the NAc in response to sucrose ↑		[109]
	6-OHDA * lesioned rats	Firing rate of phasic response in the STN ↑		[110]
	6-OHDA * lesioned mice	Spontaneous and evoked firing rate in the STN ↑	**M**: Optogenetic inhibition of STN neurons Optogenetic stimulation of STN neurons **E**: Mechanical and thermal allodynia ↓	[111]

* PSL: Partial Sciatic nerve Ligation, SNI: Spared Nerve Injury, CCI: Chronic Constriction Injury, CPN: Common Peroneal Nerve, SNL: Spinal Nerve Ligation, CFA: Complete Freund’s Adjuvant, 6-OHDA: 6-hydroxydopamine, EA: Electroacupuncture, GB30: Acupuncture point indicating the gluteal region, GB34: Acupuncture point indicating the midline of the lateral thigh, ZIP: Blocker of LTP maintenance, PCC0208009: IDO1 inhibitor, Rapamycin: mTOR inhibitor, Ro25681: NR2B inhibitor, Hyperoside: Flavonoid compound isolated from a folk remedy, CP96345: NK1 receptor antagonist, M617: a GalR1 agonist, SRT1720: Selective SIRT1 agonist. Arrows up (↑) and down (↓) indicate ‘increase’ and ‘decrease’, respectively.

## Data Availability

Not applicable.
